# Supplementary material for: Weighted Cox regression for the prediction of heterogeneous patient subgroups
Source: BMC Med Inform Decis Mak. 2021 Dec 7;21:342. doi: 10.1186/s12911-021-01698-1 (PMC8650299; doi:10.1186/s12911-021-01698-1)
Supplement: Supplementary file 1 — Additional file 1: Additional supporting information referenced in the Results Sections: Description of the NSCLC data preprocessing, Supplementary Figures 1-6, Supplementary Tables 1-2). [file 12911_2021_1698_MOESM1_ESM.pdf]

Supporting information for "Weighted Cox regression for the prediction of heterogeneous patient subgroups"

by Katrin Madjar and Jörg Rahnenführer

Preprocessing of NSCLC cohorts

Four non-small cell lung cancer (NSCLC) cohorts with overall survival and censoring information, clinical pathologic information, and Affymetrix gene expression data of the tumor material, were downloaded from the Gene Expression Omnibus (GEO) data repository (Edgar et al., 2002) and manually curated as follows. Raw gene expression data (CEL-files), measured on the Affymetrix HG-U133 Plus 2.0 array, were normalized using frozen robust multiarray analysis (fRMA) (McCall et al., 2010). All cohorts were checked for duplicates by looking at correlations of the expression value vectors. Duplicates, small cell cancer samples and normal (non-tumorous) samples, as well as samples with missing survival endpoint were removed. More details on the data curation process can be found in Hellwig et al. (2016). The resulting four NSCLC cohorts comprise $n = 635$ patients with available overall survival endpoint, clinical variables (age at time of diagnosis, sex, pTNM stage, histology and smoking status), and gene expression data: GSE29013 ($n = 55$, 18 events), GSE31210 ($n = 226$, 35 events), GSE37745 ($n = 194$, 143 events), and GSE50081 ($n = 160$, 65 events). A summary of clinical pathologic variables of all cohorts is presented in Supplementary Table 1.

The total number of measured genetic covariates (probe sets representing genes) in each cohort is 54675. For the majority of these probe sets the expression values are noisy and do not contain relevant information regarding survival outcome. This makes the identification of the prognostic genes more difficult and increases computation time. Therefore, besides, we use two preselected reduced gene sets for analysis. One subset is defined by the 1000 features (probe sets) with the highest variance in gene expression values across all four cohorts, referred to as top-1000-variance genes. This selection is based on the assumption that important prognostic genes imply systematic changes in their expression values and thus, a larger variance compared to irrelevant noise genes. The second subset is a literature-based selection of 14 prognostic genes from Kratz et al. (2012) and 20 prognostic gene signatures from a systematic literature review and meta-analysis-based evaluation by Tang et al. (2017). The review by Tang et al. (2017) includes 42 published gene signatures derived

from genome-wide mRNA gene expression studies, whereof 17 and 8 signatures, respectively, were found to be prognostic in the histological types adenocarcinoma and squamous cell carcinoma (a total of 20 different signatures as 5 signatures are prognostic in both histological types). We ignore how the genes were combined numerically in the original signatures (using statistical models) and combine only single genes with the 14 genes from Kratz et al. (2012) to one prognostic gene list. We translate gene symbols into corresponding probe set IDs of the Affymetrix HG-U133 Plus 2.0 array using the R/Bioconductor annotation package `hgu133plus2.db` (Carlson, 2016). Not all genes have a match on this array. Therefore, we use a reduced prognostic gene list for analysis comprising 3429 different probe sets that are related to 1323 different genes.

Supplementary Figures

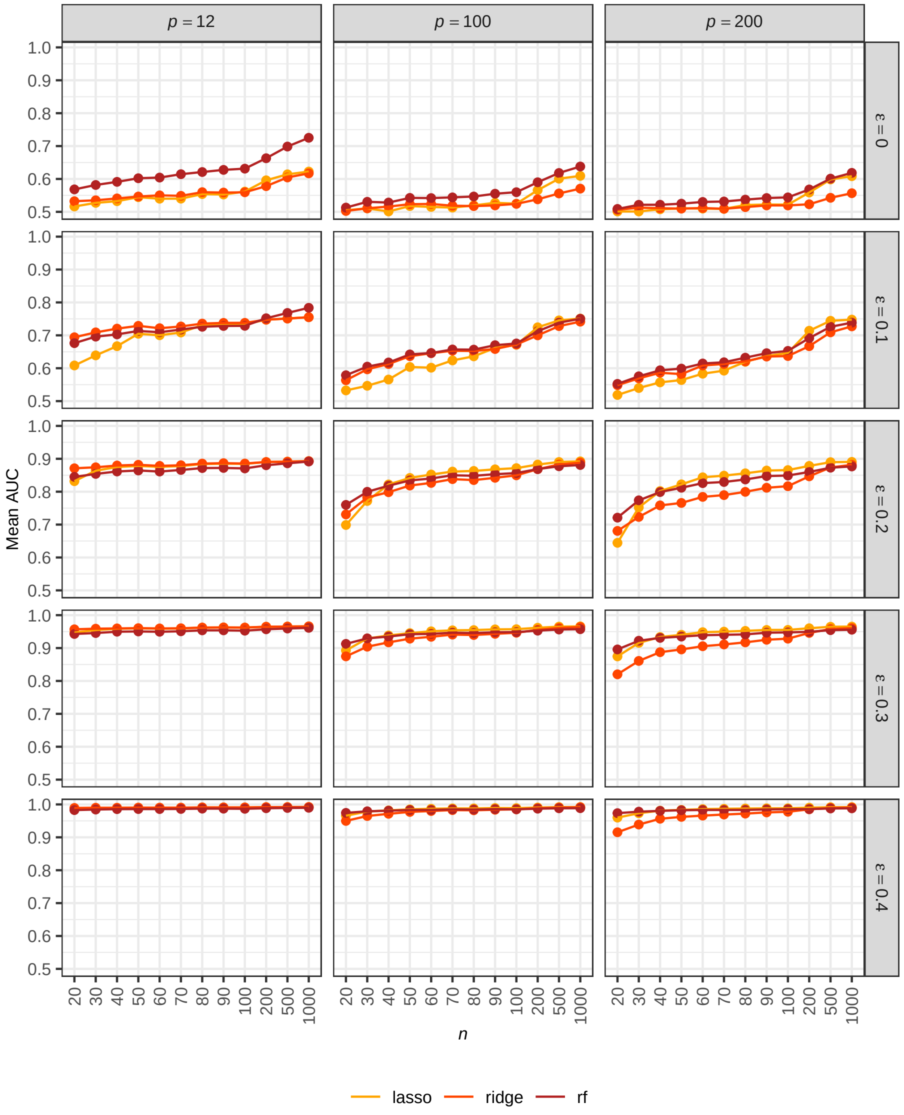

Figure 1: Mean AUC (averaged across all test sets) for the different classification methods (colors) and simulation scenarios.

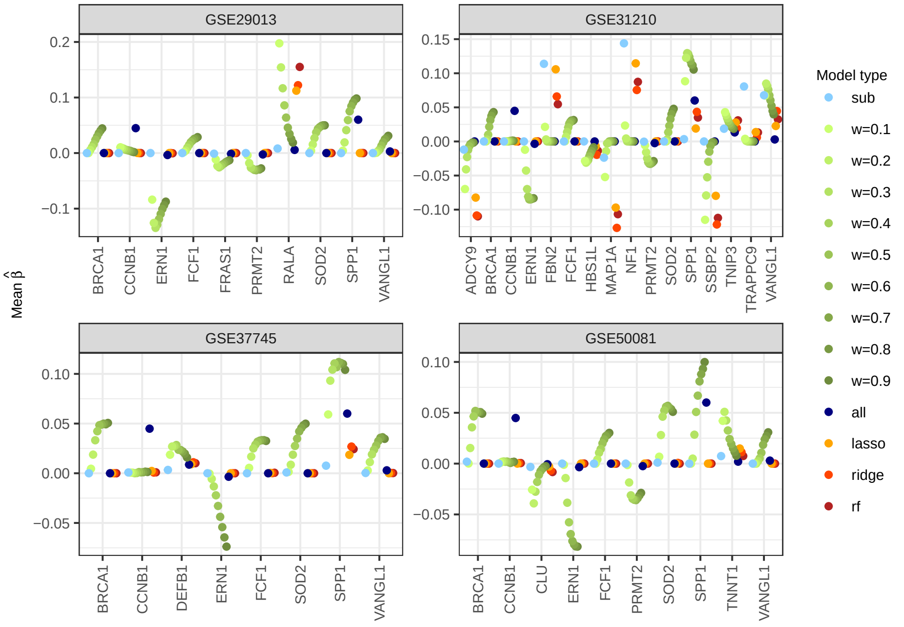

Figure 2: Mean estimated regression coefficients (averaged across all training sets) of selected genes in all Cox models including the prognostic genes as co-variates. For each subgroup genes with a mean inclusion frequency larger or equal than 0.5 in any model type are selected.

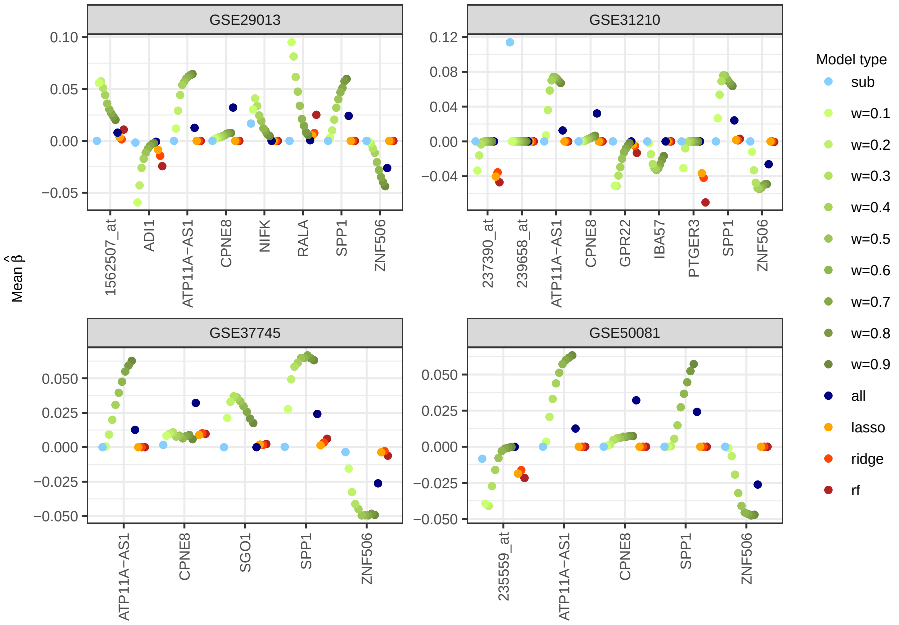

Figure 3: Mean estimated regression coefficients (averaged across all training sets) of selected genes in all Cox models including all genes as covariates. For each subgroup genes with a mean inclusion frequency larger or equal than 0.5 in any model type are selected.

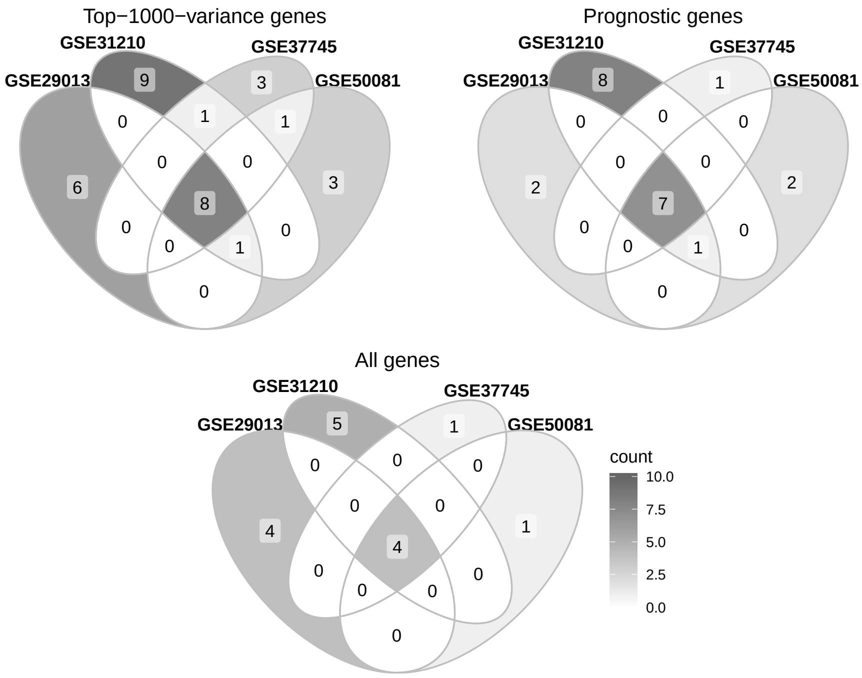

Figure 4: Venn diagram of the number of selected genes in all subgroup Cox models including as covariates the top-1000-variance genes, prognostic genes, or all genes. For each gene set and subgroup, genes with a mean inclusion frequency larger or equal than 0.5 in any model type are selected.

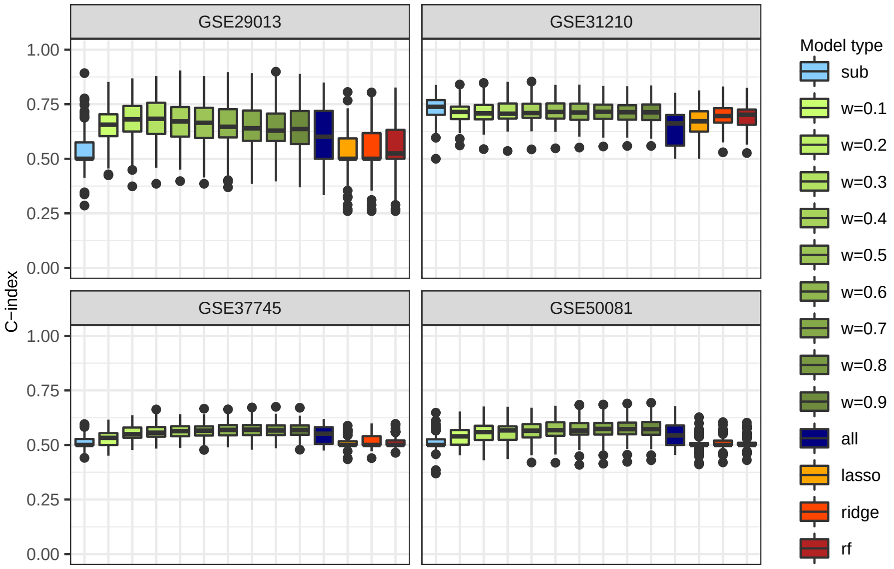

Figure 5: Boxplots of C-index based on all test sets for the prediction of each subgroup under different types of Cox models including the prognostic genes.

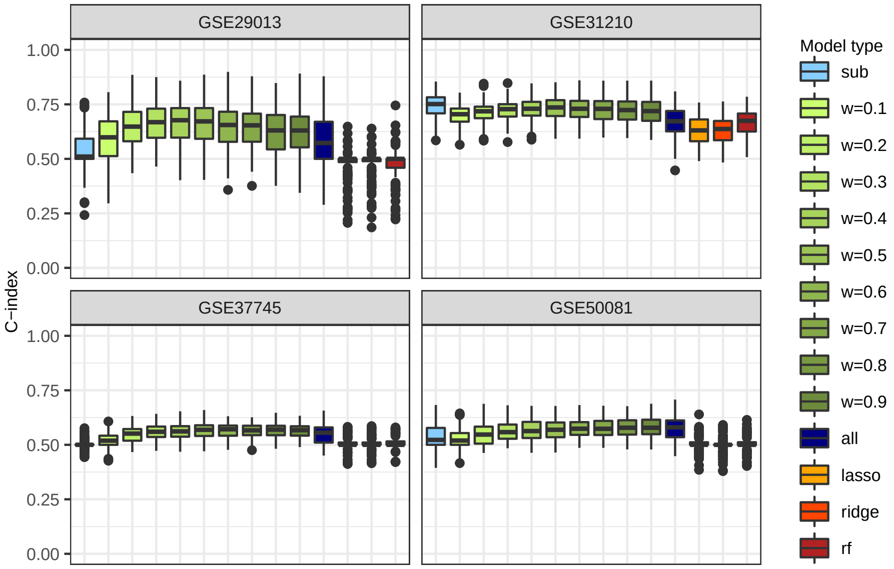

Figure 6: Boxplots of C-index based on all test sets for the prediction of each subgroup under different types of Cox models including all genes.

Supplementary Tables

Table 1: Summary of clinical pathologic variables of all NSCLC cohorts. Absolute frequencies of variable values.

Variable	Values	GSE29013	GSE31210	GSE37745	GSE50081
Sample size		55	226	194	160
Age (years)	min.	32	30	39	40
	mean	64	60	64	68
	max.	76	76	84	87
Sex	male	38	105	105	88
	female	17	121	89	72
pTNM stage	I	24	168	128	112
	II-IV	31	58	66	48
Histology	SQC	25	0	64	35
	ADC	30	226	106	115
	other NSCLC	0	0	24	10
Smoking status	never-smoker	2	115	15	24
	current/former smoker	53	111	179	136
Survival status	censoring	37	191	51	95
	event	18	35	143	65

Table 2: Mean C-index, averaged across all test data sets and subgroups, for all 252 simulation scenarios and all 14 Cox model types

ϵ	p	n	sub	$w = 0.1$	$w = 0.2$	$w = 0.3$	$w = 0.4$	$w = 0.5$	$w = 0.6$	$w = 0.7$	$w = 0.8$	$w = 0.9$	all	lasso	ridge	rf
**0**																
	12	20	0.69	0.76	0.78	0.78	0.78	0.77	0.77	0.77	0.77	0.77	0.76	0.76	0.76	0.76
		30	0.75	0.81	0.81	0.81	0.80	0.80	0.79	0.79	0.79	0.79	0.78	0.78	0.78	0.78
		40	0.80	0.83	0.82	0.81	0.81	0.80	0.80	0.79	0.79	0.78	0.78	0.78	0.78	0.78
		50	0.82	0.84	0.83	0.82	0.82	0.81	0.80	0.80	0.79	0.79	0.79	0.79	0.79	0.79
		60	0.84	0.84	0.83	0.82	0.82	0.81	0.80	0.80	0.80	0.79	0.79	0.79	0.79	0.80
		70	0.85	0.84	0.83	0.83	0.82	0.81	0.81	0.80	0.80	0.79	0.79	0.79	0.79	0.80
		80	0.85	0.85	0.84	0.83	0.83	0.82	0.81	0.81	0.80	0.80	0.80	0.80	0.80	0.81
		90	0.85	0.85	0.84	0.83	0.82	0.81	0.81	0.80	0.80	0.80	0.79	0.79	0.79	0.80
		100	0.86	0.85	0.84	0.83	0.82	0.82	0.81	0.80	0.80	0.80	0.79	0.79	0.79	0.80
		200	0.87	0.86	0.85	0.84	0.83	0.82	0.81	0.81	0.80	0.80	0.80	0.80	0.80	0.82
		500	0.87	0.86	0.85	0.84	0.83	0.82	0.82	0.81	0.81	0.80	0.80	0.80	0.80	0.83
		1000	0.87	0.86	0.85	0.84	0.83	0.83	0.82	0.81	0.81	0.80	0.80	0.81	0.81	0.83
	100	20	0.56	0.62	0.65	0.66	0.66	0.67	0.67	0.67	0.67	0.67	0.65	0.66	0.67	0.66
		30	0.59	0.69	0.72	0.73	0.74	0.73	0.73	0.73	0.73	0.73	0.72	0.72	0.73	0.72
		40	0.64	0.73	0.76	0.76	0.76	0.76	0.76	0.75	0.75	0.75	0.74	0.75	0.74	0.74
		50	0.68	0.77	0.78	0.79	0.78	0.78	0.78	0.77	0.77	0.77	0.77	0.76	0.77	0.77
		60	0.74	0.79	0.80	0.80	0.79	0.79	0.78	0.78	0.78	0.77	0.77	0.77	0.77	0.77
		70	0.76	0.80	0.81	0.80	0.80	0.79	0.79	0.78	0.78	0.78	0.77	0.77	0.77	0.77
		80	0.79	0.81	0.81	0.81	0.80	0.80	0.79	0.79	0.79	0.78	0.78	0.78	0.78	0.78
		90	0.80	0.82	0.82	0.81	0.80	0.80	0.80	0.79	0.79	0.78	0.78	0.78	0.78	0.78
		100	0.82	0.83	0.82	0.82	0.81	0.81	0.80	0.80	0.79	0.79	0.79	0.79	0.79	0.79
		200	0.86	0.85	0.84	0.83	0.82	0.81	0.81	0.80	0.80	0.80	0.79	0.79	0.79	0.80
		500	0.87	0.86	0.85	0.84	0.83	0.82	0.81	0.81	0.81	0.80	0.80	0.80	0.80	0.80
		1000	0.87	0.86	0.85	0.84	0.83	0.82	0.82	0.81	0.81	0.80	0.80	0.80	0.80	0.81

	200	20	0.54	0.59	0.62	0.63	0.64	0.64	0.65	0.65	0.65	0.65	0.64	0.65	0.65	0.65
		30	0.57	0.65	0.68	0.70	0.71	0.71	0.71	0.71	0.71	0.71	0.70	0.70	0.70	0.70
		40	0.60	0.70	0.73	0.74	0.75	0.74	0.74	0.74	0.74	0.73	0.73	0.73	0.73	0.73
		50	0.64	0.74	0.77	0.77	0.77	0.77	0.76	0.76	0.76	0.75	0.75	0.75	0.75	0.75
		60	0.68	0.76	0.78	0.78	0.78	0.77	0.77	0.77	0.76	0.76	0.76	0.76	0.76	0.76
		70	0.73	0.79	0.80	0.80	0.79	0.79	0.78	0.78	0.78	0.77	0.77	0.77	0.77	0.77
		80	0.76	0.80	0.80	0.80	0.80	0.79	0.79	0.78	0.78	0.78	0.77	0.77	0.77	0.77
		90	0.78	0.81	0.81	0.80	0.80	0.79	0.79	0.79	0.78	0.78	0.78	0.78	0.78	0.78
		100	0.80	0.82	0.81	0.81	0.80	0.80	0.79	0.79	0.79	0.78	0.78	0.78	0.78	0.78
		200	0.85	0.84	0.83	0.82	0.82	0.81	0.81	0.80	0.80	0.79	0.79	0.79	0.79	0.79
		500	0.87	0.86	0.85	0.84	0.83	0.82	0.81	0.81	0.80	0.80	0.80	0.80	0.80	0.80
		1000	0.87	0.86	0.85	0.84	0.83	0.82	0.82	0.81	0.81	0.80	0.80	0.80	0.80	0.80
**0.1**																
	12	20	0.68	0.76	0.77	0.78	0.77	0.77	0.77	0.77	0.76	0.76	0.76	0.76	0.77	0.77
		30	0.76	0.82	0.82	0.81	0.81	0.80	0.80	0.79	0.79	0.78	0.78	0.79	0.79	0.80
		40	0.80	0.83	0.82	0.82	0.81	0.80	0.80	0.79	0.79	0.78	0.78	0.79	0.80	0.80
		50	0.82	0.84	0.83	0.83	0.82	0.81	0.80	0.80	0.79	0.79	0.79	0.80	0.80	0.81
		60	0.84	0.85	0.84	0.83	0.82	0.81	0.81	0.80	0.80	0.79	0.79	0.80	0.81	0.81
		70	0.85	0.85	0.84	0.83	0.82	0.81	0.81	0.80	0.80	0.79	0.79	0.81	0.81	0.81
		80	0.85	0.85	0.85	0.84	0.83	0.82	0.81	0.81	0.80	0.80	0.80	0.82	0.82	0.82
		90	0.85	0.85	0.84	0.84	0.83	0.82	0.81	0.81	0.80	0.80	0.79	0.82	0.82	0.82
		100	0.86	0.86	0.85	0.84	0.83	0.82	0.81	0.81	0.80	0.80	0.79	0.82	0.82	0.82
		200	0.87	0.86	0.85	0.84	0.83	0.83	0.82	0.81	0.81	0.80	0.80	0.83	0.83	0.83
		500	0.87	0.87	0.86	0.85	0.84	0.83	0.82	0.82	0.81	0.80	0.80	0.84	0.84	0.84
		1000	0.87	0.87	0.86	0.85	0.84	0.83	0.82	0.82	0.81	0.81	0.80	0.84	0.84	0.85
	100	20	0.56	0.61	0.64	0.66	0.66	0.67	0.67	0.67	0.67	0.67	0.66	0.67	0.67	0.66
		30	0.59	0.68	0.72	0.73	0.73	0.73	0.73	0.73	0.73	0.73	0.72	0.72	0.72	0.72
		40	0.63	0.73	0.76	0.76	0.76	0.76	0.76	0.75	0.75	0.75	0.74	0.74	0.75	0.75
		50	0.69	0.77	0.78	0.79	0.78	0.78	0.78	0.77	0.77	0.77	0.77	0.77	0.77	0.77
		60	0.73	0.79	0.80	0.80	0.79	0.79	0.78	0.78	0.78	0.77	0.77	0.77	0.77	0.77

		70	0.76	0.80	0.81	0.80	0.80	0.79	0.79	0.78	0.78	0.78	0.77	0.78	0.78	0.78
		80	0.79	0.82	0.82	0.81	0.81	0.80	0.79	0.79	0.79	0.78	0.78	0.79	0.79	0.79
		90	0.80	0.82	0.82	0.81	0.81	0.80	0.79	0.79	0.79	0.78	0.78	0.79	0.79	0.79
		100	0.82	0.83	0.83	0.82	0.81	0.81	0.80	0.80	0.79	0.79	0.79	0.79	0.79	0.79
		200	0.86	0.85	0.84	0.83	0.82	0.82	0.81	0.80	0.80	0.80	0.79	0.81	0.81	0.80
		500	0.87	0.86	0.85	0.84	0.83	0.82	0.82	0.81	0.81	0.80	0.80	0.82	0.82	0.82
		1000	0.87	0.87	0.86	0.85	0.84	0.83	0.82	0.81	0.81	0.80	0.80	0.83	0.83	0.82
	200	20	0.53	0.59	0.62	0.63	0.64	0.65	0.65	0.65	0.66	0.65	0.63	0.65	0.66	0.65
		30	0.57	0.65	0.68	0.70	0.71	0.71	0.71	0.71	0.71	0.71	0.70	0.71	0.70	0.70
		40	0.60	0.70	0.73	0.74	0.74	0.74	0.74	0.74	0.74	0.73	0.73	0.73	0.73	0.73
		50	0.64	0.74	0.76	0.77	0.77	0.77	0.76	0.76	0.76	0.75	0.75	0.75	0.75	0.75
		60	0.67	0.77	0.78	0.78	0.78	0.77	0.77	0.77	0.76	0.76	0.76	0.76	0.76	0.76
		70	0.73	0.79	0.80	0.79	0.79	0.79	0.78	0.78	0.77	0.77	0.77	0.77	0.77	0.77
		80	0.76	0.80	0.81	0.80	0.80	0.79	0.79	0.78	0.78	0.78	0.77	0.78	0.78	0.78
		90	0.78	0.81	0.81	0.80	0.80	0.79	0.79	0.79	0.78	0.78	0.78	0.78	0.78	0.78
		100	0.80	0.82	0.82	0.81	0.80	0.80	0.79	0.79	0.79	0.78	0.78	0.78	0.78	0.78
		200	0.85	0.84	0.84	0.83	0.82	0.81	0.81	0.80	0.80	0.79	0.79	0.81	0.80	0.80
		500	0.87	0.86	0.85	0.84	0.83	0.82	0.82	0.81	0.81	0.80	0.80	0.82	0.81	0.81
		1000	0.87	0.87	0.86	0.85	0.83	0.83	0.82	0.81	0.81	0.80	0.80	0.83	0.82	0.82
**0.2**																
	12	20	0.68	0.75	0.77	0.77	0.77	0.77	0.77	0.77	0.76	0.76	0.75	0.78	0.79	0.78
		30	0.76	0.81	0.81	0.81	0.80	0.80	0.79	0.79	0.79	0.78	0.78	0.82	0.82	0.81
		40	0.80	0.83	0.82	0.81	0.81	0.80	0.79	0.79	0.79	0.78	0.78	0.82	0.83	0.82
		50	0.82	0.84	0.83	0.82	0.81	0.81	0.80	0.80	0.79	0.79	0.79	0.84	0.84	0.83
		60	0.84	0.84	0.83	0.82	0.81	0.81	0.80	0.80	0.79	0.79	0.79	0.84	0.84	0.83
		70	0.85	0.85	0.84	0.83	0.82	0.81	0.81	0.80	0.80	0.79	0.79	0.85	0.84	0.84
		80	0.85	0.85	0.84	0.83	0.82	0.82	0.81	0.81	0.80	0.80	0.80	0.85	0.85	0.84
		90	0.85	0.85	0.84	0.83	0.82	0.82	0.81	0.80	0.80	0.80	0.79	0.85	0.85	0.84
		100	0.86	0.86	0.84	0.83	0.82	0.82	0.81	0.80	0.80	0.80	0.79	0.85	0.85	0.84
		200	0.87	0.86	0.85	0.84	0.83	0.82	0.82	0.81	0.80	0.80	0.80	0.86	0.86	0.85

		500	0.87	0.87	0.86	0.85	0.84	0.83	0.82	0.81	0.81	0.80	0.80	0.87	0.86	0.86
		1000	0.87	0.87	0.86	0.85	0.84	0.83	0.82	0.81	0.81	0.80	0.80	0.87	0.87	0.86
	100	20	0.56	0.61	0.64	0.65	0.66	0.67	0.67	0.67	0.67	0.67	0.66	0.67	0.67	0.67
		30	0.60	0.68	0.71	0.72	0.73	0.73	0.73	0.73	0.73	0.73	0.72	0.73	0.73	0.73
		40	0.64	0.72	0.75	0.76	0.76	0.75	0.75	0.75	0.75	0.75	0.74	0.76	0.75	0.75
		50	0.69	0.76	0.78	0.78	0.78	0.78	0.77	0.77	0.77	0.77	0.76	0.78	0.78	0.78
		60	0.73	0.78	0.79	0.79	0.79	0.78	0.78	0.78	0.77	0.77	0.77	0.80	0.79	0.78
		70	0.77	0.80	0.80	0.80	0.79	0.79	0.78	0.78	0.78	0.77	0.77	0.80	0.79	0.79
		80	0.79	0.81	0.81	0.80	0.80	0.79	0.79	0.79	0.78	0.78	0.78	0.81	0.80	0.80
		90	0.80	0.82	0.81	0.80	0.80	0.79	0.79	0.79	0.78	0.78	0.78	0.82	0.80	0.80
		100	0.82	0.83	0.82	0.81	0.81	0.80	0.80	0.79	0.79	0.79	0.79	0.82	0.81	0.81
		200	0.86	0.85	0.84	0.83	0.82	0.81	0.81	0.80	0.80	0.79	0.79	0.84	0.83	0.82
		500	0.87	0.86	0.85	0.84	0.83	0.82	0.81	0.81	0.80	0.80	0.80	0.86	0.86	0.83
		1000	0.87	0.87	0.86	0.84	0.83	0.82	0.82	0.81	0.81	0.80	0.80	0.86	0.86	0.84
	200	20	0.53	0.59	0.62	0.63	0.64	0.64	0.65	0.65	0.65	0.65	0.63	0.65	0.65	0.66
		30	0.56	0.64	0.68	0.69	0.70	0.71	0.71	0.71	0.71	0.70	0.70	0.70	0.70	0.71
		40	0.60	0.68	0.72	0.73	0.74	0.74	0.74	0.74	0.73	0.73	0.73	0.74	0.74	0.74
		50	0.65	0.73	0.76	0.76	0.76	0.76	0.76	0.75	0.75	0.75	0.75	0.76	0.76	0.76
		60	0.68	0.76	0.77	0.77	0.77	0.77	0.76	0.76	0.76	0.76	0.76	0.78	0.77	0.77
		70	0.73	0.78	0.79	0.79	0.78	0.78	0.78	0.77	0.77	0.77	0.77	0.79	0.78	0.78
		80	0.76	0.79	0.80	0.79	0.79	0.79	0.78	0.78	0.78	0.77	0.77	0.80	0.79	0.78
		90	0.78	0.80	0.80	0.80	0.79	0.79	0.78	0.78	0.78	0.78	0.78	0.81	0.79	0.79
		100	0.80	0.81	0.81	0.80	0.80	0.79	0.79	0.78	0.78	0.78	0.78	0.81	0.80	0.79
		200	0.85	0.84	0.83	0.82	0.81	0.81	0.80	0.80	0.79	0.79	0.79	0.84	0.82	0.81
		500	0.87	0.86	0.85	0.84	0.83	0.82	0.81	0.81	0.80	0.80	0.80	0.86	0.85	0.83
		1000	0.87	0.87	0.85	0.84	0.83	0.82	0.82	0.81	0.80	0.80	0.80	0.86	0.86	0.83
**0.3**																
	12	20	0.68	0.75	0.76	0.77	0.77	0.77	0.77	0.77	0.77	0.77	0.76	0.79	0.79	0.80
		30	0.76	0.80	0.80	0.80	0.80	0.80	0.79	0.79	0.79	0.78	0.78	0.83	0.83	0.83

	40	0.80	0.82	0.81	0.81	0.80	0.80	0.79	0.79	0.79	0.78	0.78	0.84	0.84	0.83
	50	0.83	0.83	0.82	0.81	0.81	0.80	0.80	0.79	0.79	0.79	0.79	0.85	0.85	0.84
	60	0.84	0.84	0.83	0.82	0.81	0.80	0.80	0.80	0.79	0.79	0.79	0.85	0.85	0.84
	70	0.85	0.84	0.83	0.82	0.81	0.81	0.80	0.80	0.79	0.79	0.79	0.86	0.86	0.85
	80	0.85	0.85	0.84	0.83	0.82	0.81	0.81	0.80	0.80	0.80	0.80	0.86	0.86	0.85
	90	0.85	0.85	0.84	0.83	0.82	0.81	0.81	0.80	0.80	0.79	0.79	0.86	0.86	0.85
	100	0.86	0.85	0.84	0.83	0.82	0.81	0.81	0.80	0.80	0.79	0.79	0.86	0.86	0.85
	200	0.87	0.86	0.85	0.83	0.83	0.82	0.81	0.81	0.80	0.80	0.80	0.87	0.87	0.86
	500	0.87	0.86	0.85	0.84	0.83	0.82	0.82	0.81	0.81	0.80	0.80	0.87	0.87	0.87
	1000	0.87	0.87	0.85	0.84	0.83	0.82	0.82	0.81	0.81	0.80	0.80	0.87	0.87	0.87
100	20	0.56	0.61	0.63	0.65	0.66	0.67	0.67	0.67	0.67	0.67	0.66	0.66	0.67	0.67
	30	0.60	0.66	0.70	0.71	0.72	0.72	0.72	0.72	0.72	0.72	0.71	0.73	0.73	0.73
	40	0.64	0.71	0.74	0.75	0.75	0.75	0.75	0.75	0.74	0.74	0.74	0.76	0.76	0.76
	50	0.69	0.75	0.77	0.77	0.77	0.77	0.77	0.77	0.77	0.77	0.76	0.79	0.78	0.78
	60	0.74	0.77	0.78	0.78	0.78	0.78	0.77	0.77	0.77	0.77	0.77	0.81	0.80	0.79
	70	0.77	0.79	0.79	0.79	0.78	0.78	0.78	0.78	0.77	0.77	0.77	0.82	0.80	0.80
	80	0.79	0.80	0.80	0.80	0.79	0.79	0.79	0.78	0.78	0.78	0.78	0.83	0.81	0.81
	90	0.80	0.81	0.80	0.80	0.79	0.79	0.79	0.78	0.78	0.78	0.78	0.83	0.82	0.81
	100	0.82	0.82	0.81	0.80	0.80	0.80	0.79	0.79	0.79	0.79	0.79	0.84	0.83	0.81
	200	0.86	0.84	0.83	0.82	0.81	0.81	0.80	0.80	0.80	0.79	0.79	0.86	0.85	0.83
	500	0.87	0.86	0.84	0.83	0.82	0.81	0.81	0.80	0.80	0.80	0.80	0.87	0.87	0.85
	1000	0.87	0.86	0.85	0.84	0.83	0.82	0.81	0.81	0.80	0.80	0.80	0.87	0.87	0.86
200	20	0.54	0.58	0.61	0.63	0.63	0.65	0.65	0.65	0.65	0.65	0.62	0.64	0.65	0.65
	30	0.56	0.63	0.67	0.69	0.70	0.70	0.70	0.70	0.70	0.70	0.70	0.70	0.70	0.71
	40	0.60	0.67	0.71	0.72	0.73	0.73	0.73	0.73	0.73	0.73	0.72	0.74	0.74	0.74
	50	0.65	0.71	0.74	0.75	0.75	0.75	0.75	0.75	0.75	0.75	0.74	0.77	0.76	0.76
	60	0.68	0.74	0.76	0.76	0.76	0.76	0.76	0.76	0.76	0.76	0.75	0.79	0.77	0.77
	70	0.73	0.77	0.77	0.78	0.78	0.77	0.77	0.77	0.77	0.77	0.77	0.80	0.79	0.78
	80	0.76	0.78	0.78	0.78	0.78	0.78	0.78	0.77	0.77	0.77	0.77	0.81	0.79	0.79
	90	0.78	0.79	0.79	0.79	0.78	0.78	0.78	0.78	0.78	0.77	0.77	0.82	0.80	0.79

		100	0.80	0.80	0.80	0.79	0.79	0.79	0.78	0.78	0.78	0.78	0.78	0.83	0.80	0.80
		200	0.85	0.84	0.82	0.81	0.81	0.80	0.80	0.79	0.79	0.79	0.79	0.85	0.83	0.82
		500	0.87	0.86	0.84	0.83	0.82	0.81	0.81	0.80	0.80	0.80	0.80	0.87	0.86	0.84
		1000	0.87	0.86	0.85	0.84	0.83	0.82	0.81	0.81	0.80	0.80	0.80	0.87	0.87	0.85
**0.4**																
	12	20	0.68	0.74	0.76	0.76	0.77	0.76	0.77	0.77	0.77	0.77	0.76	0.79	0.79	0.79
		30	0.75	0.80	0.80	0.79	0.79	0.79	0.79	0.79	0.79	0.79	0.78	0.84	0.84	0.83
		40	0.80	0.81	0.81	0.80	0.80	0.79	0.79	0.79	0.79	0.78	0.78	0.84	0.84	0.84
		50	0.83	0.82	0.81	0.81	0.80	0.80	0.80	0.79	0.79	0.79	0.79	0.85	0.85	0.84
		60	0.84	0.83	0.82	0.81	0.80	0.80	0.80	0.79	0.79	0.79	0.79	0.86	0.86	0.85
		70	0.85	0.83	0.82	0.81	0.81	0.80	0.80	0.80	0.79	0.79	0.79	0.86	0.86	0.85
		80	0.85	0.84	0.83	0.82	0.82	0.81	0.81	0.80	0.80	0.80	0.80	0.87	0.87	0.86
		90	0.85	0.84	0.83	0.82	0.81	0.81	0.80	0.80	0.80	0.79	0.79	0.86	0.86	0.86
		100	0.86	0.84	0.83	0.82	0.81	0.81	0.80	0.80	0.80	0.79	0.79	0.87	0.87	0.86
		200	0.87	0.85	0.84	0.83	0.82	0.81	0.81	0.81	0.80	0.80	0.80	0.87	0.87	0.87
		500	0.87	0.86	0.84	0.83	0.82	0.82	0.81	0.81	0.81	0.80	0.80	0.87	0.87	0.87
		1000	0.87	0.86	0.84	0.83	0.83	0.82	0.81	0.81	0.81	0.80	0.80	0.87	0.87	0.87
	100	20	0.56	0.59	0.63	0.65	0.66	0.66	0.66	0.67	0.67	0.67	0.65	0.66	0.67	0.67
		30	0.59	0.65	0.69	0.71	0.72	0.72	0.72	0.72	0.72	0.72	0.71	0.72	0.73	0.73
		40	0.63	0.69	0.73	0.74	0.74	0.74	0.74	0.74	0.74	0.74	0.73	0.76	0.75	0.76
		50	0.69	0.73	0.76	0.76	0.76	0.77	0.76	0.76	0.76	0.76	0.76	0.79	0.78	0.78
		60	0.73	0.75	0.77	0.77	0.77	0.77	0.77	0.77	0.77	0.77	0.76	0.81	0.79	0.79
		70	0.76	0.77	0.78	0.78	0.78	0.77	0.77	0.77	0.77	0.77	0.77	0.82	0.80	0.80
		80	0.79	0.79	0.79	0.79	0.79	0.78	0.78	0.78	0.78	0.78	0.78	0.83	0.82	0.81
		90	0.80	0.79	0.79	0.79	0.79	0.78	0.78	0.78	0.78	0.78	0.78	0.83	0.82	0.81
		100	0.82	0.80	0.80	0.80	0.79	0.79	0.79	0.79	0.79	0.79	0.78	0.84	0.83	0.82
		200	0.85	0.84	0.82	0.81	0.81	0.80	0.80	0.80	0.79	0.79	0.79	0.86	0.85	0.84
		500	0.87	0.85	0.84	0.83	0.82	0.81	0.81	0.80	0.80	0.80	0.80	0.87	0.87	0.86
		1000	0.87	0.86	0.84	0.83	0.82	0.81	0.81	0.81	0.80	0.80	0.80	0.87	0.87	0.86

	200	20	0.54	0.58	0.60	0.62	0.63	0.64	0.64	0.64	0.65	0.64	0.63	0.63	0.65	0.65
		30	0.57	0.62	0.66	0.68	0.69	0.70	0.70	0.70	0.70	0.70	0.69	0.69	0.70	0.71
		40	0.60	0.66	0.70	0.71	0.72	0.72	0.73	0.73	0.73	0.72	0.72	0.74	0.73	0.74
		50	0.64	0.70	0.73	0.74	0.74	0.74	0.74	0.74	0.74	0.74	0.74	0.77	0.75	0.76
		60	0.68	0.72	0.74	0.75	0.75	0.75	0.75	0.75	0.75	0.75	0.75	0.78	0.76	0.77
		70	0.73	0.75	0.76	0.77	0.77	0.77	0.77	0.77	0.77	0.76	0.76	0.81	0.78	0.78
		80	0.76	0.76	0.77	0.77	0.77	0.77	0.77	0.77	0.77	0.77	0.77	0.81	0.79	0.79
		90	0.77	0.77	0.78	0.78	0.78	0.78	0.78	0.77	0.77	0.77	0.77	0.82	0.80	0.79
		100	0.80	0.78	0.78	0.78	0.78	0.78	0.78	0.78	0.78	0.77	0.77	0.83	0.81	0.80
		200	0.85	0.83	0.81	0.81	0.80	0.80	0.79	0.79	0.79	0.79	0.79	0.85	0.84	0.82
		500	0.87	0.85	0.83	0.82	0.82	0.81	0.80	0.80	0.80	0.80	0.80	0.87	0.87	0.85
		1000	0.87	0.86	0.84	0.83	0.82	0.81	0.81	0.80	0.80	0.80	0.80	0.87	0.87	0.85
**0.5**																
	12	20	0.69	0.73	0.75	0.76	0.76	0.77	0.77	0.77	0.76	0.77	0.75	0.78	0.79	0.79
		30	0.76	0.78	0.79	0.79	0.79	0.79	0.79	0.79	0.79	0.78	0.78	0.84	0.84	0.83
		40	0.80	0.80	0.80	0.80	0.79	0.79	0.79	0.79	0.79	0.78	0.78	0.84	0.84	0.84
		50	0.82	0.82	0.81	0.80	0.80	0.80	0.80	0.79	0.79	0.79	0.78	0.85	0.85	0.85
		60	0.84	0.82	0.81	0.81	0.80	0.80	0.80	0.79	0.79	0.79	0.79	0.86	0.86	0.85
		70	0.85	0.83	0.82	0.81	0.81	0.80	0.80	0.80	0.79	0.79	0.79	0.86	0.86	0.85
		80	0.85	0.84	0.82	0.82	0.81	0.81	0.81	0.80	0.80	0.80	0.80	0.87	0.87	0.86
		90	0.85	0.83	0.82	0.81	0.81	0.80	0.80	0.80	0.80	0.79	0.79	0.86	0.86	0.86
		100	0.86	0.84	0.82	0.82	0.81	0.81	0.80	0.80	0.80	0.79	0.79	0.87	0.87	0.86
		200	0.87	0.85	0.83	0.82	0.82	0.81	0.81	0.80	0.80	0.80	0.80	0.87	0.87	0.87
		500	0.87	0.85	0.84	0.83	0.82	0.82	0.81	0.81	0.80	0.80	0.80	0.87	0.87	0.87
		1000	0.87	0.85	0.84	0.83	0.82	0.82	0.81	0.81	0.81	0.80	0.80	0.87	0.87	0.87
	100	20	0.56	0.59	0.62	0.64	0.65	0.66	0.66	0.66	0.66	0.66	0.65	0.65	0.67	0.66
		30	0.59	0.65	0.68	0.70	0.71	0.71	0.72	0.71	0.72	0.71	0.70	0.71	0.72	0.72
		40	0.64	0.68	0.72	0.73	0.74	0.74	0.74	0.74	0.73	0.73	0.73	0.75	0.74	0.75
		50	0.69	0.72	0.75	0.76	0.76	0.76	0.76	0.76	0.76	0.76	0.76	0.78	0.77	0.78
		60	0.74	0.74	0.76	0.76	0.77	0.77	0.77	0.77	0.76	0.76	0.76	0.80	0.79	0.79

		70	0.76	0.76	0.77	0.77	0.77	0.77	0.77	0.77	0.77	0.77	0.77	0.82	0.80	0.80
		80	0.79	0.77	0.78	0.78	0.78	0.78	0.78	0.78	0.78	0.77	0.77	0.83	0.81	0.81
		90	0.80	0.78	0.78	0.78	0.78	0.78	0.78	0.78	0.78	0.78	0.77	0.83	0.82	0.81
		100	0.82	0.79	0.79	0.79	0.79	0.79	0.79	0.79	0.79	0.78	0.78	0.84	0.82	0.82
		200	0.85	0.83	0.82	0.81	0.80	0.80	0.80	0.80	0.79	0.79	0.79	0.86	0.85	0.84
		500	0.87	0.84	0.83	0.82	0.81	0.81	0.80	0.80	0.80	0.80	0.80	0.87	0.87	0.86
		1000	0.87	0.85	0.83	0.83	0.82	0.81	0.81	0.80	0.80	0.80	0.80	0.87	0.87	0.86
	200	20	0.54	0.57	0.60	0.62	0.63	0.64	0.64	0.64	0.64	0.64	0.63	0.62	0.64	0.64
		30	0.56	0.61	0.65	0.67	0.69	0.69	0.70	0.70	0.70	0.70	0.68	0.68	0.70	0.70
		40	0.60	0.65	0.69	0.71	0.71	0.72	0.72	0.72	0.72	0.72	0.71	0.72	0.73	0.73
		50	0.64	0.68	0.72	0.73	0.73	0.74	0.74	0.74	0.74	0.74	0.73	0.76	0.74	0.75
		60	0.68	0.70	0.73	0.74	0.75	0.75	0.75	0.75	0.75	0.75	0.75	0.78	0.75	0.76
		70	0.73	0.73	0.75	0.76	0.76	0.76	0.76	0.76	0.76	0.76	0.76	0.80	0.78	0.78
		80	0.76	0.75	0.76	0.77	0.77	0.77	0.77	0.77	0.77	0.76	0.76	0.81	0.79	0.79
		90	0.78	0.76	0.77	0.77	0.77	0.77	0.77	0.77	0.77	0.77	0.77	0.82	0.79	0.79
		100	0.80	0.77	0.78	0.78	0.78	0.78	0.78	0.77	0.77	0.77	0.77	0.83	0.80	0.80
		200	0.85	0.82	0.81	0.80	0.80	0.79	0.79	0.79	0.79	0.79	0.79	0.85	0.84	0.83
		500	0.87	0.84	0.83	0.82	0.81	0.81	0.80	0.80	0.80	0.80	0.80	0.87	0.87	0.85
		1000	0.87	0.85	0.83	0.82	0.82	0.81	0.81	0.80	0.80	0.80	0.80	0.87	0.87	0.86
**1**																
	12	20	0.68	0.72	0.74	0.76	0.76	0.76	0.77	0.77	0.76	0.77	0.75	0.76	0.76	0.77
		30	0.75	0.77	0.78	0.78	0.78	0.78	0.78	0.78	0.78	0.78	0.77	0.81	0.82	0.83
		40	0.80	0.79	0.79	0.79	0.79	0.79	0.79	0.78	0.78	0.78	0.78	0.83	0.83	0.84
		50	0.82	0.80	0.80	0.80	0.80	0.79	0.79	0.79	0.79	0.79	0.78	0.85	0.84	0.85
		60	0.84	0.80	0.80	0.80	0.80	0.80	0.79	0.79	0.79	0.79	0.79	0.85	0.85	0.86
		70	0.85	0.81	0.81	0.80	0.80	0.80	0.80	0.80	0.79	0.79	0.79	0.86	0.86	0.86
		80	0.85	0.82	0.81	0.81	0.81	0.81	0.80	0.80	0.80	0.80	0.80	0.87	0.86	0.87
		90	0.85	0.81	0.81	0.81	0.80	0.80	0.80	0.80	0.80	0.79	0.79	0.86	0.86	0.86
		100	0.86	0.82	0.81	0.81	0.80	0.80	0.80	0.80	0.80	0.79	0.79	0.87	0.86	0.86
		200	0.87	0.83	0.82	0.81	0.81	0.81	0.80	0.80	0.80	0.80	0.80	0.87	0.87	0.87

	500	0.87	0.83	0.82	0.82	0.81	0.81	0.81	0.81	0.80	0.80	0.80	0.87	0.87	0.87
	1000	0.87	0.83	0.83	0.82	0.82	0.81	0.81	0.81	0.80	0.80	0.80	0.87	0.87	0.87
100	20	0.56	0.58	0.60	0.62	0.64	0.65	0.65	0.65	0.65	0.65	0.64	0.62	0.65	0.62
	30	0.59	0.62	0.65	0.68	0.69	0.69	0.69	0.70	0.70	0.69	0.68	0.66	0.70	0.66
	40	0.64	0.64	0.69	0.70	0.71	0.71	0.72	0.72	0.72	0.71	0.71	0.70	0.70	0.70
	50	0.69	0.68	0.72	0.73	0.73	0.74	0.74	0.74	0.74	0.74	0.73	0.73	0.73	0.73
	60	0.73	0.70	0.72	0.74	0.74	0.74	0.74	0.74	0.74	0.74	0.74	0.75	0.74	0.75
	70	0.77	0.71	0.74	0.74	0.75	0.75	0.75	0.75	0.75	0.75	0.75	0.76	0.75	0.76
	80	0.79	0.73	0.75	0.75	0.76	0.76	0.76	0.76	0.76	0.76	0.76	0.78	0.76	0.78
	90	0.80	0.74	0.75	0.76	0.76	0.76	0.76	0.76	0.76	0.76	0.76	0.79	0.77	0.79
	100	0.82	0.75	0.76	0.77	0.77	0.77	0.78	0.78	0.77	0.77	0.77	0.80	0.79	0.80
	200	0.86	0.80	0.80	0.80	0.79	0.79	0.79	0.79	0.79	0.79	0.79	0.85	0.83	0.84
	500	0.87	0.82	0.82	0.81	0.81	0.80	0.80	0.80	0.80	0.80	0.80	0.87	0.87	0.87
	1000	0.87	0.83	0.82	0.82	0.81	0.81	0.81	0.80	0.80	0.80	0.80	0.87	0.87	0.87
200	20	0.54	0.56	0.59	0.60	0.62	0.63	0.63	0.63	0.64	0.64	0.62	0.59	0.63	0.60
	30	0.56	0.59	0.63	0.66	0.67	0.68	0.68	0.68	0.68	0.69	0.68	0.64	0.68	0.65
	40	0.60	0.62	0.66	0.68	0.69	0.70	0.70	0.70	0.70	0.70	0.69	0.68	0.70	0.68
	50	0.64	0.64	0.69	0.70	0.71	0.71	0.72	0.72	0.72	0.72	0.71	0.70	0.72	0.71
	60	0.68	0.67	0.70	0.72	0.72	0.72	0.72	0.73	0.73	0.73	0.72	0.72	0.73	0.72
	70	0.73	0.70	0.72	0.73	0.73	0.74	0.74	0.74	0.74	0.74	0.74	0.74	0.73	0.74
	80	0.76	0.71	0.72	0.73	0.74	0.74	0.74	0.74	0.74	0.74	0.74	0.75	0.74	0.75
	90	0.77	0.72	0.73	0.74	0.75	0.75	0.75	0.75	0.75	0.75	0.75	0.76	0.75	0.76
	100	0.80	0.73	0.74	0.75	0.75	0.75	0.75	0.75	0.75	0.75	0.75	0.77	0.76	0.76
	200	0.85	0.78	0.78	0.78	0.78	0.78	0.78	0.78	0.78	0.78	0.78	0.83	0.81	0.82
	500	0.87	0.82	0.81	0.81	0.80	0.80	0.80	0.80	0.80	0.80	0.80	0.87	0.85	0.85
	1000	0.87	0.83	0.82	0.81	0.81	0.81	0.80	0.80	0.80	0.80	0.80	0.87	0.87	0.87

References

Carlson, M. (2016). *hgu133plus2.db: Affymetrix Human Genome U133 Plus 2.0 Array annotation data (chip hgu133plus2).* R package version 3.2.3.

Edgar, R., Domrachev, M., and Lash, A. E. (2002). Gene Expression Omnibus: NCBI gene expression and hybridization array data repository. *Nucleic Acids Research* **30,** 207–210.

Hellwig, B., Madjar, K., Edlund, K., Marchan, R., Cadenas, C., Heimes, A.-S., Almstedt, K., Lebrecht, A., Sicking, I., Battista, M. J., Micke, P., Schmidt, M., Hengstler, J. G., and Rahnenführer, J. (2016). Epsin Family Member 3 and Ribosome-Related Genes Are Associated with Late Metastasis in Estrogen Receptor-Positive Breast Cancer and Long-Term Survival in Non-Small Cell Lung Cancer Using a Genome-Wide Identification and Validation Strategy. *PLOS ONE* **11,** e0167585.

Kratz, J. R., He, J., Van Den Eeden, S. K., Zhu, Z.-H., Gao, W., Pham, P. T., Mulvihill, M. S., Ziaei, F., Zhang, H., Su, B., Zhi, X., Quesenberry, C. P., Habel, L. A., Deng, Q., Wang, Z., Zhou, J., Li, H., Huang, M.-C., Yeh, C.-C., Segal, M. R., Ray, M. R., Jones, K. D., Raz, D. J., Xu, Z., Jahan, T. M., Berryman, D., He, B., Mann, M. J., and Jablons, D. M. (2012). A practical molecular assay to predict survival in resected non-squamous, non-small-cell lung cancer: development and international validation studies. *The Lancet* **379,** 823–832.

McCall, M. N., Bolstad, B. M., and Irizarry, R. A. (2010). Frozen robust multiarray analysis (fRMA). *Biostatistics* **11,** 242–253.

Tang, H., Wang, S., Xiao, G., Schiller, J., Papadimitrakopoulou, V., Minna, J., Wistuba, I. I., and Xie, Y. (2017). Comprehensive evaluation of published gene expression prognostic signatures for biomarker-based lung cancer clinical studies. *Annals of Oncology* **28,** 733–740.
